# Being Moved by the Self and Others: Influence of Empathy on Self-Motion Perception

**DOI:** 10.1371/journal.pone.0048293

**Published:** 2013-01-11

**Authors:** Christophe Lopez, Caroline J. Falconer, Fred W. Mast

**Affiliations:** 1 Department of Psychology, University of Bern, Bern, Switzerland; 2 Laboratoire de Neurosciences Intégratives et Adaptatives, Centre National de la Recherche Scientifique (CNRS), Aix-Marseille Université, Marseille, France; 3 Center for Cognition, Learning and Memory, University of Bern, Bern, Switzerland; University of Bologna, Italy

## Abstract

**Background:**

The observation of conspecifics influences our bodily perceptions and actions: Contagious yawning, contagious itching, or empathy for pain, are all examples of mechanisms based on resonance between our own body and others. While there is evidence for the involvement of the mirror neuron system in the processing of motor, auditory and tactile information, it has not yet been associated with the perception of self-motion.

**Methodology/Principal Findings:**

We investigated whether viewing our own body, the body of another, and an object in motion influences self-motion perception. We found a visual-vestibular congruency effect for self-motion perception when observing self and object motion, and a reduction in this effect when observing someone else's body motion. The congruency effect was correlated with empathy scores, revealing the importance of empathy in mirroring mechanisms.

**Conclusions/Significance:**

The data show that vestibular perception is modulated by agent-specific mirroring mechanisms. The observation of conspecifics in motion is an essential component of social life, and self-motion perception is crucial for the distinction between the self and the other. Finally, our results hint at the presence of a “vestibular mirror neuron system”.

## Introduction

Self-motion perception is crucial for the survival of the species to distinguish between one's own body motion and the motion of the external world, including conspecifics and objects located around us. Various studies have demonstrated the contribution of vestibular [1]–[5], visual [6]–[10], and somatosensory signals [11], [12] to self-motion perception. Optic flow moving in a given direction is known to induce illusory self-motion and to elicit strong postural reactions [6], [8]. Similarly, moving sounds evoke sensations of self-motion in otherwise stable listeners (review in [13]). More recently, virtual reality has been used to increase the feeling of presence in immersive visual-auditory environments and to manipulate self-motion perception (e.g. [14], [15]). Although low-level visual, vestibular and somatosensory contributions to self-motion perception have been studied for over a century, studies have ignored how observing the motion of our conspecifics can influence self-motion perception. This is particularly surprising given the fact that exposures to large crowds in cities, or the observation of movements in recreational activities such as ballet dancing and the practice of sport, are very common experiences.

In this study, we investigate whether the observation of conspecifics can influence self-motion perception. The present research question is motivated by the importance of shared body representations between the self and others [16], whereby one's sensory and emotional states are modulated by the observation of another's body. Prototypical examples are contagious yawning [17] and contagious itching [18]. The resonance between the self and others has been well described for the motor system: Observing another body performing an action facilitates the execution of this action [19], [20], an effect mediated by a “mirror neuron system” in the human brain [21]–[23]. Self-other resonance following similar principles has also been described for sensory and emotional processing. For example, observing another body being touched activates the secondary somatosensory cortex [24] and facilitates the detection of tactile stimuli applied to one's own body [25]–[27]. Similarly, observing another person experiencing painful stimuli activates pain networks in the observer's brain [28], [29]. Given that social interactions involve the observation of other bodies in motion, the current experiment was designed to determine whether the perception of others in motion contains information that can ultimately influence self-motion perception. Recent neuroimaging studies showed that observing videos of full-bodies in motion activated sensorimotor networks likely active during the execution of body motion [30], [31]. However, to date, no study has investigated whether observing passive full-body motions can influence the detection of one's own full body passive motion. Accordingly, we developed a new experimental paradigm combining a state-of-the-art vestibular platform and visual stimulation to investigate how self-, other- and object-related visual information influences self-motion perception. Observers were seated on a motion platform and passively rotated in the yaw plane [4]. They were asked to indicate in which direction (left/right) they were rotated while looking at videos depicting their own body, another body, or an object rotating in the yaw plane. The spatial congruency between self-motion and the item displayed in the video was manipulated by creating congruent trials (specular congruency) and incongruent trials (non-specular congruency).

## Materials and Methods

### Participants

In this study 21 healthy volunteers participated (10 females and 11 males, mean age 27± SD 4 years). All participants were right-handed except one, as confirmed by the Edinburgh Handedness inventory [32]. Participants had normal or corrected-to-normal vision and declared no history of vestibular, neurological, or psychiatric disease. Experimental procedures were approved by the local Ethics Committee (University of Bern) and followed the ethical recommendations laid down in the Declaration of Helsinki. All participants gave written informed consent.

### Self-motion stimuli

**Figure 1:**
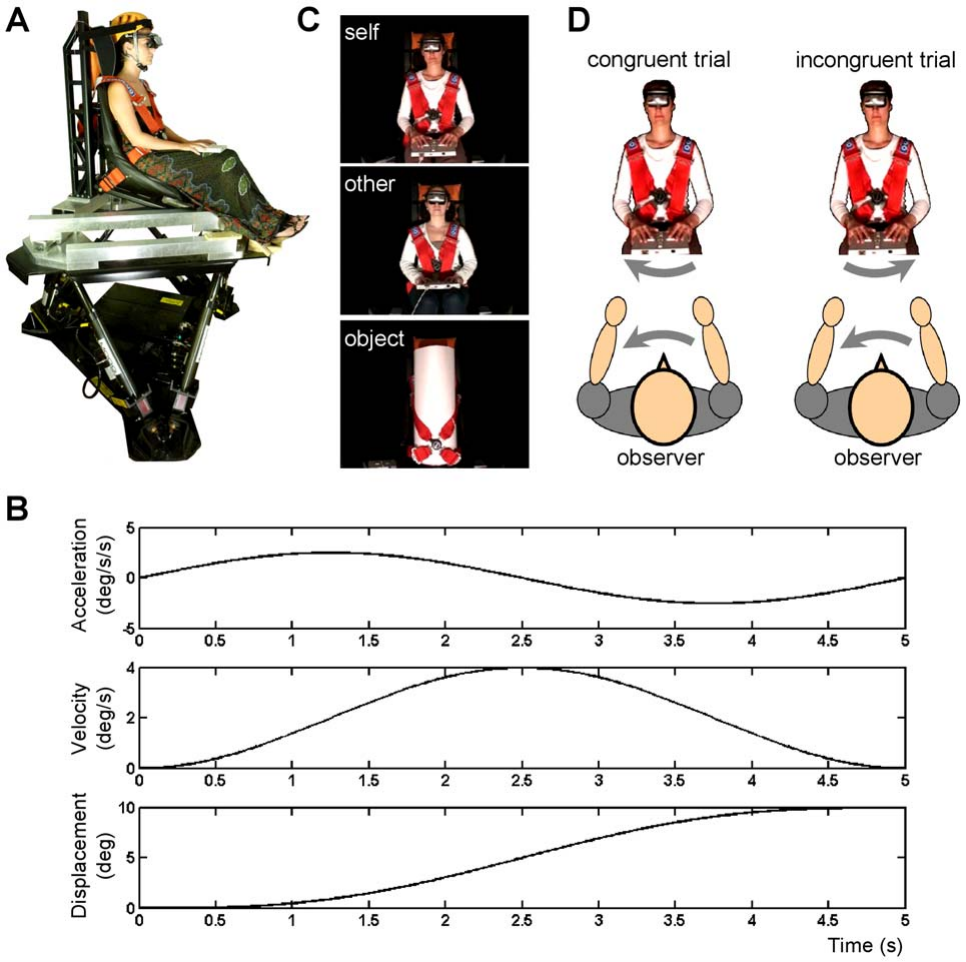
Experimental setup. (**A**) Self-motion perception was tested in 21 observers seated on a motion platform. Motion stimuli were yaw rotations lasting for 5 s with peak velocity of 0.1°/s, 0.6°/s, 1.1°/s, and 4°/s. (**B**) Example of a motion profile consisting of a single cycle sinusoidal acceleration. Acceleration, velocity and displacement are illustrated for the highest velocity used in this experiment (4°/s). (**C**) Observers wore a head-mounted display through which 5-s videos were presented, depicting their own body, the body of another participant matched for gender and age, or an inanimate object. (**D**) During congruent trials the observers and the object depicted in the video were rotated in the same direction (specular congruency). The participants depicted in the photographs have given written informed consent, as outlined in the PLoS consent form, to publication of their photograph.

Motion stimuli were generated using a six degrees of freedom motion platform (MOOG 6DOF2000E) (**Figure 1A**). Motion profiles were single cycle sinusoidal accelerations in the yaw plane (**Figure 1B**), chosen on the basis that they mimic natural human kinetics [4]. Yaw rotations refer to rotations in the horizontal plane around the longitudinal body axis, which is the main vertical axis going from the head to the feet (e.g., shaking the head from right to left, as if to say “no”, would be a yaw rotation of the head). A pilot test was conducted to determine the appropriate four motion profiles that would incorporate peak velocities around and above the vestibular threshold (in accordance with [33]). Nine participants took part in the pilot test, which consisted of nine motion profiles with peak velocities ranging from 0.25°/s to 4.25°/s in increments of 0.50°/s. For this pilot test, participants wore a head-mounted display through which they were presented a frontal picture of the chair mounted on the MOOG platform. The participants' task was to indicate as quickly and as accurately as possible whether their own body was moved to the right or to the left by means of a response pad. Participants were instructed to press a button with their right index finger if the perceived self-motion was directed to their right. Conversely, they had to press a button with their left index finger if the perceived self-motion was directed to the left. Each of the motion profiles had 10 repetitions, and response times and the percentage of correct responses were calculated. Motion profiles with a peak velocity of 0.1°/s, 0.6°/s, 1.1°/s, and 4°/s were then selected on the basis of the pilot study to use in the experiment proper (systematic sampling in accordance with [33]).

### Visual stimuli

Visual stimuli consisted of three categories of videos. Videos were either of the participant (self video), another unknown participant (age- and sex-matched; other video), or a cylindrical white object located on the same motion platform (object video) (**Figure 1C**). Videos of the participants (self videos) were recorded before the experiment proper. For that, participants were seated, secured, and made comfortable in the chair mounted on the motion platform. A video camera was located 1.4 m in front of the participants and captured the motion of most of the participant's body seated on the chair (from the head to the knees). All visual references in the background were excluded by a black curtain mounted vertically behind the motion platform. Video recordings were then taken of the participants being rotated to their left and to their right around their longitudinal body axis during a 5-s sinusoidal motion profile with a peak velocity of 4°/s. Videos of the ‘other’ participant were pre-recorded using similar procedures, i.e. during passive rotations of an actor around its main longitudinal body axis and with the same motion profile (duration: 5 s; velocity: 4°/s). During the recordings, the actor was also wearing the same head-mounted display as the participants did during the experiment proper. A male actor (who did not take part in the experiment as a participant) was used to create the videos depicting another unknown male body, presented to all male participants. Likewise, a female actor was used to create the videos depicting an unknown female body, and subsequently shown to all the female participants. Thus, the ‘other’ videos were kept constant for male and female participants. The unknown body seen in the video was age-matched because our participants were all within the same age range. Videos of the cylindrical object were pre-recorded using similar procedures, i.e. during yaw rotations with the same motion profile (duration: 5 s; velocity: 4°/s). All videos were mirror-reversed and cropped to a standardized image size using Adobe Premiere Pro CS5.5. Visual stimuli were presented via a high resolution (800×600 pixels) head-mounted display (Z800 3DVisor, eMagin) with a 40° field of view, which the participants also wore during the initial video recording.

### Experimental procedures

Participants were initially introduced to the MOOG motion platform for pre-experiment proper video recordings. While these videos were being edited, participants dismounted the chair and were further instructed about the task. After video editing, participants were relocated back to the chair, the head-mounted display was put on, and their head was secured with a head strap to minimize movement during the experiment. Participants also wore headphones emitting white noise to eliminate any potential motion cues from auditory signals. An additional head band was placed around the head-mounted display to eliminate any external visual cues to self-motion.

Self, other, and object videos were presented in the head-mounted display using SuperLab 4 software, which was triggered by the motion profiles programmed by the MOOG system. Thus, the onset of motion profiles was synchronous to visual stimuli. Participants were told to watch the videos presented in the head-mounted display and to indicate as quickly and as accurately as possible whether their own body was moved to the right or to the left. They were told that the video and the self-motion profile would last five seconds, but response time was not restricted. Responses were given using a response pad (RB-520, Cedrus Corporation, San Pedro, Ca, USA) with their index fingers. Participants were instructed to press a button with their right index finger if the perceived self-motion was directed to their right. Conversely, they had to press a button with their left index finger if the perceived self-motion was directed to the left.

The experiment proper was a 3 *Video* (type of video shown in the head-mounted display: self, other, object) ×2 *Motion Congruency* (congruent vs. incongruent motion direction of the video and the motion platform) ×4 *Velocity* (angular velocity of the motion platform: 0.1°/s, 0.6°/s, 1.1°/s, and 4°/s) design, with 16 repetitions of each of the 24 stimulus combinations. Thus, each participant completed 384 trials. In order to create conflict between vestibular and visual signals, we manipulated the congruency between the direction of the observer's body rotation and that of the object seen in the video (**Figure 1D**). During congruent trials the observers and the object depicted in the video were rotated in the same direction (specular congruency), whereas during incongruent trials the observers and the object in the video were rotated in a non-specular way. Forty-eight trials were randomly allocated to one of eight blocks. These eight blocks were then randomized across participants. Participants took short breaks after each block. Before the experiment proper, participants also completed a training session consisting of a random selection of 20 trials for familiarization with the response pad and the experimental procedures.

### Empathy questionnaire

Participants completed the empathy questionnaire developed by Baron-Cohen and Wheelwright [34]. This questionnaire has been designed to calculate an Empathy Quotient (EQ) gauging individual empathy traits by means of statements pertaining to three subscales: cognitive empathy, emotional reactivity, and social skills [35]. For example, the cognitive empathy scale asked participants to rate the extent to which they agree or disagree with the statement *“I can tell if someone is masking their true emotion”*; The emotional reactivity scale asked participants to rate the extent to which they agree or disagree with the statement *“I tend to get emotionally involved with a friend's problems”*; The social skills scale asked participants to rate the extent to which they agree or disagree with the statement *“I don't tend to find social situations confusing”*. Ratings were completed on a four-point scale ranging from “Strongly disagree” to “Strongly agree”. EQ performance can be represented as an all encompassing score or individual subscale scores. Total EQ scores have been shown to correlate with performance during perspective taking tasks [36] and subscale scores have been shown to selectively correlate with tactile perception in mirror-touch synesthetes [37].

### Data analysis

We calculated the mean response time for correct answers and mean percentage of correct answers for each combination of motion velocity, video and congruency. We used an arcsine-square root transformation of the percentage of correct answers according to previous psychophysical experiments [38]–[42]. Trials yielding incorrect answers were discarded from the analysis of the response times. In the present experiment, we focused on response times, which have been shown to be more sensitive than accuracy to reveal cross-modal conflicts [43]–[45]. We also calculated a congruency effect (CE) adapted from the cross-modal congruency effect used to investigate visual-tactile conflicts [43]–[45]. Individual CEs were calculated as the difference in response times between the incongruent trials and congruent trials and they were correlated with individual scores from the empathy questionnaire.

## Results

### Response times

**Figure 2:**
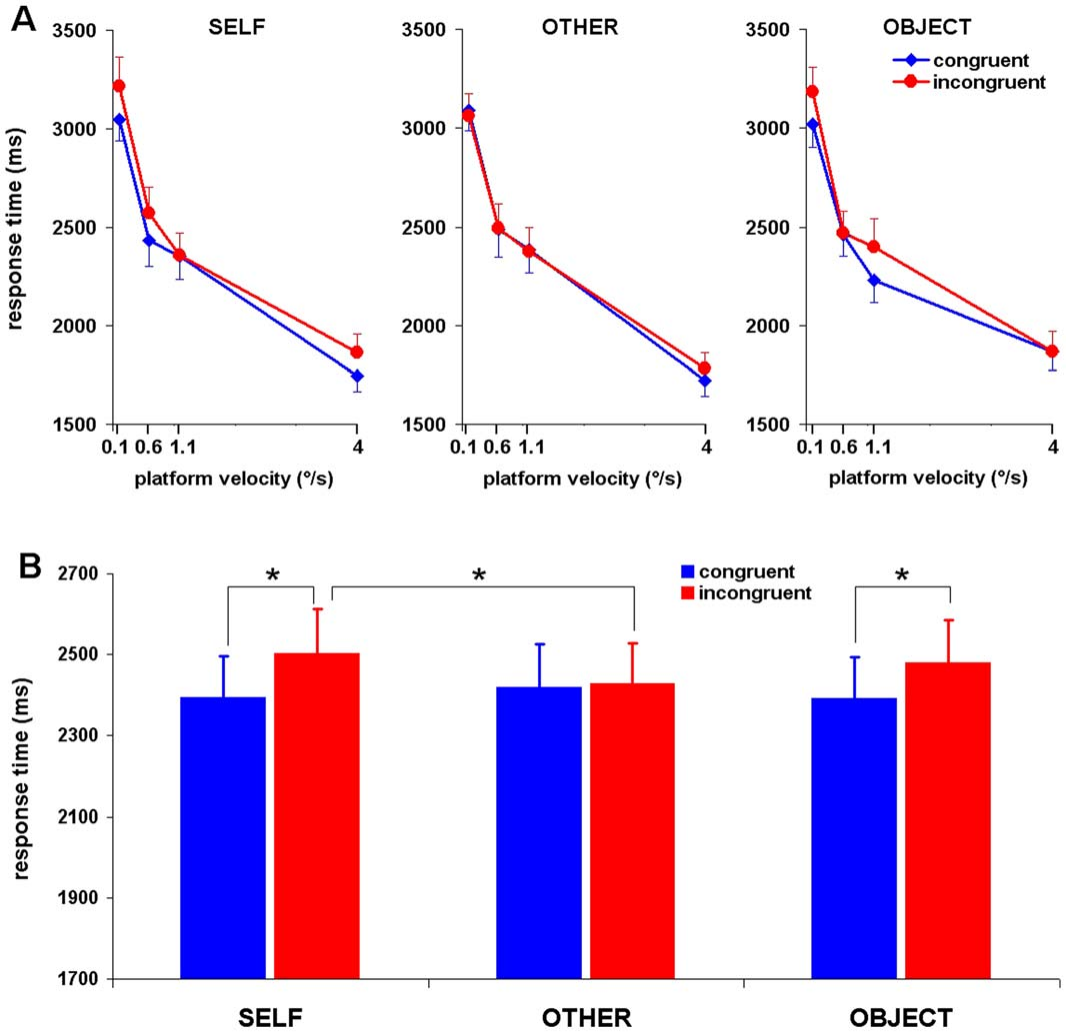
Average response times for correct answers. (**A**) Mean response time as a function of the type of video seen, the velocity of the motion platform, and the congruency of the motion depicted in the video. (**B**) Modulation of self-motion perception by the social content of the video: Significant interaction of Video×Motion congruency for the response times. * denotes statistical significance (two-tailed paired *t*-tests, *P*<0.05). Vertical bars depict SEM.

The average response time was analyzed using repeated-measures ANOVA with the within-subject factors: Velocity (angular velocity of the motion platform: 0.1, 0.6, 1.1 and 4°/s), Motion Congruency (congruent, incongruent), and Video (self, other, object). Results revealed a significant main effect of Velocity (*F*_3,60_ = 205, *P*<0.001), with response times being shorter for higher angular velocities of the motion platform (see **Figure 2A**). There was also a significant main effect of Motion Congruency (*F*_1,20_ = 6.77, *P* = 0.017). That is, response times were longer when self-motion and the motion displayed in the video were incongruent.

Importantly, a significant interaction of Motion congruency×Video was found (*F*_2,40_ = 3.76, *P* = 0.03), indicating that visual-vestibular congruency effects were modulated by the social information provided by the video (**Figure 2B**). The interaction reflects longer response times for incongruent trials when compared to congruent trials, for the self video (two-tailed paired *t*-test, *P* = 0.008) and object video (paired *t*-test, *P* = 0.026), but not for the other video (paired *t*-test, *P* = 0.802). The mean congruency effect (CE), calculated as the difference in response times between the incongruent and congruent trials, was 107±36 ms for the self video and 87±36 ms for the object video. Post-hoc analysis revealed that the mean CE for the other video (7±29 ms) was significantly reduced when compared to the CE for the self videos (two-tailed paired *t*-test, *P* = 0.026) and the object videos (*P* = 0.016). There was no difference between the CE for self videos and object videos (*P* = 0.64). These results indicate that the observation of one's own body motion, or the motion of an object, disrupts self-motion perception when the motion is incongruent. A post-hoc analysis of the interaction of Motion congruency×Video revealed that response times for incongruent trials when viewing the self video are significantly higher than when viewing the other video (paired *t*-test, *P*<0.05) (see **Figure 2B**). This effect is not present for the analysis of the congruent trials. This result suggests a higher order cognitive interaction between incongruent trials and the social content of the video. Thus, the analysis provides further evidence for the driving force behind the Video×Motion Congruency interaction: only when viewing another person was there no disruptive influence of incongruent trials. These results can be compared with those found by Heed et al. [44], whereby a reduction in crossmodal congruency effects for visual-tactile stimuli reflects a reduction in response times to incongurent trials during the presence of ‘another’ in the peripersonal space.

Noteworthy is the nearly significant three-way interaction between Velocity, Video, and Motion Congruency (*F*_6,120_ = 2.09, *P* = 0.058) which is illustrated in **Figure 2A**. Finally, there was no main effect of the Video (*F*_2,40_ = 0.61, *P* = 0.55) and no significant interaction of Velocity×Video (*F*_6,120_ = 1.51, *P* = 0.18) and of Velocity×Motion Congruency (*F*_3,60_ = 0.28, *P* = 0.84).

### Accuracy

**Figure 3:**
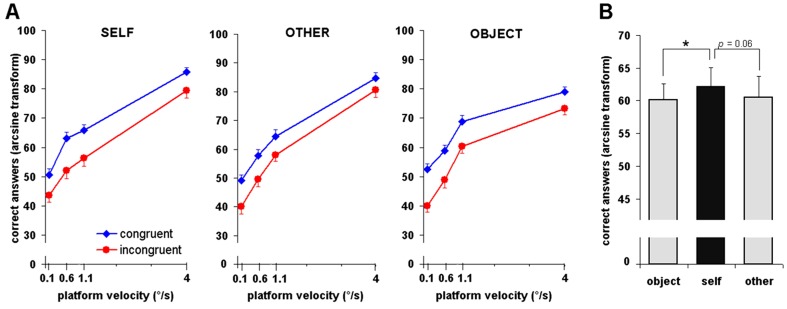
Accuracy of self-motion detection task. (**A**) Average percentage of correct answers (arcsine transformation) as a function of the type of video seen, the velocity of the motion platform, and the congruency of the motion depicted in the video. (**B**) Main effect of video. * denotes statistical significance (two-tailed paired *t*-tests, *P*<0.05). Vertical bars depict SEM.

The same repeated-measures ANOVA was run on the percentage of correct answers (arcsine transformed, see *Materials and Methods*) and revealed a significant main effect of Velocity (*F*_3,60_ = 223, *P*<0.001). As illustrated in **Figure 3A**, the performance increased with the angular velocity of the motion platform. The Motion Congruency effect was also significant (*F*_1,20_ = 12.68, *P*<0.005). The participants discriminated better their own body motion when observing a video moving congruently (i.e. in a specular way) than when observing a video moving incongruently. There was a nearly significant main effect of the Video (F*_2,40_* = 3.20, *P* = 0.05), suggesting that the information displayed in the video influences the performance (**Figure 3B**). Interestingly, post-hoc analyses (two-tailed paired *t*-tests) revealed overall better performance for self-motion perception when participants saw their own body in the video (self videos *vs* object videos: *P*<0.05; self videos *vs* other videos: *P* = 0.06), irrespective of the motion congruency. The analysis also revealed a significant interaction of Velocity×Video (*F*_6,120_ = 5.35, *P*<0.001), suggesting that task difficulty (the velocity of the platform) influenced differently self-motion perception according to the item depicted in the video. Post-hoc analyses (two-tailed paired *t*-test) showed higher accuracy for self videos than object videos for platform velocities of 0.6°/s (statistical trend: *P* = 0.06), 1.1°/s (*P*<0.05) and 4°/s (*P*<0.05), as well as higher accuracy for self videos than other videos for platform velocities of 0.6°/s (*P*<0.05). The opposite effect, i.e. higher accuracy for object videos than other videos and self videos, was found only for platform velocity of 1.1°/s (*P*<0.05). Finally, there was no interaction of Video×Motion Congruency (*F*_2,40_ = 0.74, *P* = 0.48), of Velocity×Motion Congruency (*F*_3,60_ = 1.69, *P* = 0.18), and the three-way interaction of Velocity×Video×Motion Congruency (*F*_6,120_ = 0.64, *P* = 0.70) was not significant.

### Correlations between congruency effect (CE) and empathy scores

Individual CEs for each video type were correlated with participant scores on the Empathy Quotient [35], [37] (**Table 1**). The overall EQ scores did not significantly correlate with the video content. However, scores on the Emotional Reactivity subscale were positively correlated with the CE for Other videos (*r* = 0.46, *P*<0.05) and Object videos (*r* = 0.48, *P*<0.05), while the social skills subscale was positively correlated with the CE for Object videos (*r* = 0.54, *P*<0.05). At first sight, the correlation of empathy scores with CE when seeing another person may appear counterintuitive. The mean CE is strongly reduced in this condition. However, individual CEs range from −268 ms to +223 ms. Those participants who showed higher empathy scores also showed a positive CE, albeit much less than for the self and object conditions (the range of CE is shifted to higher values for the self videos: −223 ms to +504 ms; as well as for the object videos: −233 ms to +432 ms).

**Table 1:** Summary of correlations between CE and empathy.

Empathy scores	self video	other video	object video
Emotional reactivity	*r* = 0.21, N.S.	***r*** ** = 0.46, ** ***P*** **<0.05** *	***r*** ** = 0.48, ** ***P*** **<0.05** *
Social skills	*r* = 0.23, N.S.	*r* = 0.27, N.S.	***r*** ** = 0.54, ** ***P*** **<0.05** *
Cognitive empathy	*r* = −0.08, N.S.	*r* = 0.29, N.S.	*r* = 0.32, N.S.
EQ score	*r* = 0.01, N.S.	***r*** ** = 0.39, ** ***P*** ** = 0.08 ** #	***r*** ** = 0.41, ** ***P*** ** = 0.06 ** #

N.S. = not significant;

* denotes statistical significance (*P*<0.05);

# denotes a statistical trend.

## Discussion

The key finding of this experiment shows that self-motion perception is influenced by the observation of the self and objects in motion, but, to a lesser extent when viewing others in motion.

These findings provide the first demonstration that self-motion perception is modulated by the observation of *one's own body in motion*. A first important result is that participants performed on average better (higher accuracy) when they saw their own body being moved than the body of someone else or an object being moved. This result is in line with previous studies on multimodal integration showing an advantage of seeing one's own body for various aspects of self perception. Serino et al. [27] showed that seeing one's own face being touched facilitates tactile detection at the level of the face (and this facilitation effect is stronger than the effect of seeing another person's face). Along the same line, seeing one's own body being touched increased the performance of tactile detection [46] and modulates tactile processing in the primary somatosensory cortex [47]. Other studies found enhanced interoceptive awareness when looking at one's own body in a mirror (e.g., heartbeat perception [48]) and better postural control [49]. Therefore, it is likely that self-observation involves some sensory representations in the brain that the observation of others cannot do or to a lesser extent [50].

The second new and important result concerning the observation of *one's own body in motion* is that the observation of the self in motion, in a specular way (here referred to as congruent trials), results in more efficient self-motion perception than the observation of non-specular motion (incongruent trials). Thus, our data indicate that visual-vestibular associations subserving self-motion perception operate in a specular way, instead of in an anatomical way (when rotations towards the participant's right are congruent with a rotation of the seen body towards its anatomical right side). A similar trend has been observed regarding tactile perception in mirror-touch synesthetes, a population of individuals who experience tactile sensations when observing touch applied to another's body. Indeed, most synesthetes experienced touch on their body part (e.g. right cheek) that is opposite to the touch seen on the other's corresponding body part (e.g. left cheek), as is they were looking at their own body in a mirror [37], [51]. Such effect may be related to the fact that the observation of one's own entire body in a mirror is a familiar situation. As pointed out by Banissy and colleagues [51], this effect may derive from “the fact that one's own head [and in the present experiment, one's own entire body] is only ever seen from a mirror-reflected perspective and this regularity may drive the choice of spatial frame” (p. 266). In addition, the specular effect reported here and in previous studies is in line with the finding that imitation behaviors are often performed following a specular mode.

Previous studies proposed that shared body representations between the self and others usually operate in a specular way (e.g. [37], [52]; but see [53]), so that observing *another's body in motion* may have a similar influence as the observation of one's own body in motion. While our data show similar visual-vestibular congruency effects on the accuracy of self-motion perception when viewing the self or another body in motion, another important finding from this study is that the analysis of the response times revealed a marked reduction in the CE during the observation of another body in motion. We found that the range of the CE was shifted to lower values during observation of other videos (−268 ms to +223 ms) as compared to self videos (−223 ms to +504 ms), showing a reduced impact of observing another's body on self-processing. This indicates that while a mechanism of perceptual resonance with others does exist (positive CEs are reduced but present in some participants), there appear to be higher-order cognitive processes that can modulate resonance between self and others in the present task. This is in line with the view that the mirror neuron system's tendency to simulate another person's actions and feelings is modulated by several cognitive, social and emotional factors (see [22], [29], [54], [55]). Below, we propose three mechanisms that could be involved in the modulation of self-other resonance during the self-motion perception task. *The first mechanism* is that the presence of another's body in motion modulates multisensory integration of visual and vestibular signals in a way that is different to viewing the self or objects. A recent study demonstrated that social interactions with conspecifics located in peripersonal space reduced cross-modal visual-tactile CE [44]. In fact, Heed and colleagues [44] found that a reduction in the cross-modal CE reflects a reduction in the disruptive influence of incongruent visual-tactile information. This was also true of our results: incongruent motion trials depicting another person in motion resulted in a facilitated self-motion perception when compared to incongruent trials depicting the self in motion (**Figure 2A**). In the same vein, studies on visual-tactile integration showed that observing another individual's face being touched facilitates the detection of tactile stimuli applied on one's own face, but this facilitation is weaker than that reported during the observation of one's own face being touched [27]. Thus, despite the existence of shared body representations, the influence of seeing one's own body has a stronger impact on self-perception than seeing the bodies of conspecifics located around us. Observing other-related visual information cross-modally influence vestibular processing in a different way than observing self-related visual information. *The second mechanism* involved in the decrease in the CE for other videos may require higher-order mechanisms gating the disruptive influence of viewing another body in motion. Our data suggest that mirroring others does not fully outweigh vestibular perception and that a dedicated mechanism may actually protect self-motion perception from full-blown perceptual resonance with others. Neural mechanisms inhibiting automatic imitation of others have been demonstrated, for example, in the prefrontal cortex [56]–[58]. Interestingly, verbal reports from several participants described the feeling that the person in the other video was trying to “dupe” them. It could be possible that cognitive processes underlying trust gate or re-weight the influence of other-related visual input. There is already evidence highlighting the role of social identity in tactile mirroring [26], empathy for pain [29], [54] and visceral resonance with others [59]. How higher-order cognitive processes such as social identity and trust influence low-level sensory perception is an interesting prospect for future research. *The third mechanism* involved in the modulation of self-other resonance during the self-motion perception task may be related to personality traits such as empathy. The positive correlation between empathy and visual-vestibular CE will be discussed in detail below; however it should be noted here that higher CE for other videos were associated with higher empathy scores. Therefore, empathy can modulate the ability to distinguish between self- and other whole-body motion. A similar trend has been described in mirror-touch synesthetes whereby more empathic participants were more disturbed by the observation of another's face being touched when they had to detect touch on their own face [37].

Our data also show an influence of *object observation* on self-motion perception. This is interesting given recent findings showing that the human mirror neuron system also encodes *active* movements performed by non-biological agents, such as robots [60], [61], or agents belonging to other species, such as monkeys and dogs [62]. Several other observations indicate that one's own body representations are influenced by the observation of stimuli applied to non-corporeal objects. For example, the tactile mirror system responds to touch applied to inanimate objects (e.g. rectangular geometrical objects [24]) and the pain neural network also responds to noxious stimuli applied to objects (e.g. a tomato [63]). Experiments conducted in mirror-touch synesthetes demonstrate that they also experience moderate tactile sensation during the observation of dummy body parts or objects being touched [64]. Altogether, these observations suggest that the mirroring system does not work in a pure body-specific way. The present CE found for object videos may reflect a similar mechanism for vestibular perception, with an affinity for *passive* motions of bodies and objects in the peripersonal space. It is not clear at the moment why such responses to objects movements have been developed in the human brain. One possibility is that such mechanisms help to predict and anticipate the motion of objects located in the external world.

It is important to note at this point of the discussion that the visual influence on self-motion perception reported above cannot be explained by low-level visual-vestibular congruency effects, nor by stimulus-response compatibility effects (i.e. Simon effect). Although the videos used in the present study did not include a coherent optic flow (i.e. an optokinetic stimulation) that could induce a proper illusory self-motion perception [6], [8], they included visual motion with a clear directional pattern. Motions of our own body (e.g. clockwise body rotations) are usually associated with the entire visual surrounding moving in the opposite direction (e.g. optic flow in counterclockwise direction). According to classical visual-vestibular interactions, better performance should be observed when a large part of the visual field is displaced in the *opposite* direction to the observer's motion, thus providing a synergistic visual-vestibular association [65]. Here, we observed an opposite pattern, with better performance when the seen object was rotated in the same direction as the observer (congruent trials). The decrease of performance in the case of incongruent motion indicates that the present effect cannot be attributed to low-level visual-vestibular interactions. More crucially, the fact that the type of video influences the response times, as well as the significant interaction of Motion congruency×Video for the response times, clearly indicate that the present results can be better explained by a top-down modulation of self-motion perception and vestibular perception, as found in the case of visual-tactile interactions [27]. Finally, it is conceivable that the present results could be explained by a stimulus-response compatibility effect, also referred to as the Simon effect in the literature, whereby response times increase if the location or direction of a visual stimulus does not match the location of the response key (see Lu and Proctor [66] for an overview). In the present case, we found that participants were faster and more accurate to detect self-motion to the left when they were looking at an item being rotated to their left (specular congruency), and this detection was indicated by a key press on a left button of a response pad (hence the compatibility between the response of the motion of the visual stimulation). Accordingly, it could be argued that part of the congruency effect reported above could be related to a Simon effect and to the natural tendency to respond in the direction of visual motion [66]. There is a large body of data showing a tendency to be faster and more accurate to react towards the source of a visual or an auditory stimulation when it is spatially compatible with the location of the response key. This effect has been demonstrated for the spatial location of static visual stimuli (e.g. a red LED), as well as for the direction of physically moving, or apparently moving, visual stimuli (e.g. Gabor patches) [66]–[71]. Similar effects have been reported with visual stimuli containing biological motion information such as point-like walkers [69]. However, our paradigm differs in many respects from those used to investigate the Simon effect. We think that a stimulus-response compatibility effect cannot account for the visual-vestibular congruency effect reported in the present study. *First*, the major counterargument is that response times to incongruent visual motion were modulated as a function of the stimulus properties displayed in the video (self, other, object). If the Simon effect were driving our results, it would have affected all visual conditions (i.e. when looking at self, other and object videos), which was evidently not the case. There was no difference between the response times for congruent and incongruent trials when the participants were exposed to the video showing another person (see **Figure 2B**). This means that, for instance, pressing on the left button in reaction to other videos with a leftward motion took as much time as pressing on the left button in reaction to a rightward visual motion, therefore revealing no Simon effect for the other videos. *Second*, although there is evidence that motor response can be facilitated when it is spatially congruent with the direction of visual motion (e.g. a moving dot or a Gabor patch; see [70] for an overview), there is also evidence suggesting that we should find a facilitation for responses located on the *opposite sid*e of the visual motion direction (e.g. [72],[73]). For example, Figliozzi et al. [73] have used optic flow stimuli to the right or to the left and showed that a Simon effect is present for the direction opposite to the optic flow. For instance, response times were decreased for presses on a right button when the optic flow was moved leftwards. Additionally, visual-vestibular interactions usually exhibit a facilitation of self-motion perception when the optic flow is moving in the opposite direction to self-motion [65]. *Third*, it has to be pointed out that the size of the congruency effect reported here is much larger than previously reported Simon effects. Previous studies revealed a Simon effect of around 20 ms due to incongruency of stimulus-response location and an even smaller Simon effect of about 7 ms due to dynamic visual stimulation [69]. In their review about the Simon effect, Lu and Proctor [66] concluded that “the magnitude [of the Simon effect] is more typically between 20 and 30 ms”. These rather small effects are in contrast with the present congruency effect that is much larger (107 ms for the self video and 87 ms for the object video). We conclude that the pattern of results reported here cannot be explained by a stimulus-response compatibility effect, but rather by the influence of social visual signals on self-motion perception.

We found that visual-vestibular CE correlated with emotional reactivity when viewing others in motion, lending support to the view that shared body representations between the self and others are linked to empathy abilities [37],[74]. Interestingly, the scores to the empathy questionnaire used in the present study [35] have been found to be negatively correlated with the response times to transform one's visual-spatial perspective into that of another participant [36]. This suggests that participants with high empathy scores can more easily put themselves in the shoes of another participant. Our data indicate that participants with higher empathy scores are more strongly influenced by the observation of another body or an object moving incongruently with respect to their own body. It is possible that observing a body being passively rotated may evoke an automatic third-person perspective taking that can ultimately influence vestibular perception (similar to the automatic tendency to imitate other's actions [57]). This proposition is in line with findings from behavioral studies showing that vestibular processing and visual-spatial perspective taking are closely intertwined [75]–[77]. Yet other behavioral and neuroimaging studies also revealed relations between empathy and bodily sensations. For example, scores to empathy questionnaires were found to correlate with the degree of incorporation and self-identification with another's face [78] as well as with the intensity of pain-related brain activations when observing noxious stimuli applied to another's body [28]. Interestingly, as one does not empathize with oneself, there was no correlation between empathy and CE for the self videos. The fact that CE for the object motion also correlated with empathy suggests that the role of empathy in understanding and predicting actions of others [79] may extend to the physical properties of objects in our surrounding.

The results from this study propose that self-motion information and social visual information converge in multimodal brain regions. Vestibular signals are processed mostly in the temporo-parietal junction, as well as in the insular, superior temporal, posterior parietal, and cingulate cortices [80]–[82], areas where visual and somatosensory signals indicating self-motion also converge [83]. Interestingly, these areas are known to integrate complex bodily and visual social signals that are relevant for empathic processing [84], reciprocal imitation [85],[86], body ownership [87], and visual-spatial perspective taking [88],[89]. Shared neural networks for vestibular processing and social visual processing may underpin the influence of the observation of passive whole-body and object motions on self-motion perception.

In conclusion, the present study provides evidence for vestibular resonance when viewing the self, others and objects moving within our environment, but also suggests different mechanisms when observing other people in motion. The observation of conspecifics in motion is an essential component of social life, and self-motion perception is crucial for the distinction between the self and the other. Our results hint at the presence of a *vestibular mirror neuron system* and will hopefully stimulate further research questions for basic and clinical research alike.
